# Potential and limits for rapid genetic adaptation to warming in a Great Barrier Reef coral

**DOI:** 10.1371/journal.pgen.1007220

**Published:** 2018-04-19

**Authors:** Mikhail V. Matz, Eric A. Treml, Galina V. Aglyamova, Line K. Bay

**Affiliations:** 1 University of Texas at Austin, Austin, Texas, United States of America; 2 University of Melbourne, Melbourne, Melbourne, Victoria, Australia; 3 Australian Institute of Marine Science, Townsville, Queensland, Australia; Harvard University, UNITED STATES

## Abstract

Can genetic adaptation in reef-building corals keep pace with the current rate of sea surface warming? Here we combine population genomics, biophysical modeling, and evolutionary simulations to predict future adaptation of the common coral *Acropora millepora* on the Great Barrier Reef (GBR). Genomics-derived migration rates were high (0.1–1% of immigrants per generation across half the latitudinal range of the GBR) and closely matched the biophysical model of larval dispersal. Both genetic and biophysical models indicated the prevalence of southward migration along the GBR that would facilitate the spread of heat-tolerant alleles to higher latitudes as the climate warms. We developed an individual-based metapopulation model of polygenic adaptation and parameterized it with population sizes and migration rates derived from the genomic analysis. We find that high migration rates do not disrupt local thermal adaptation, and that the resulting standing genetic variation should be sufficient to fuel rapid region-wide adaptation of *A*. *millepora* populations to gradual warming over the next 20–50 coral generations (100–250 years). Further adaptation based on novel mutations might also be possible, but this depends on the currently unknown genetic parameters underlying coral thermal tolerance and the rate of warming realized. Despite this capacity for adaptation, our model predicts that coral populations would become increasingly sensitive to random thermal fluctuations such as ENSO cycles or heat waves, which corresponds well with the recent increase in frequency of catastrophic coral bleaching events.

## Author summary

Coral reefs worldwide are suffering high mortality from severe thermal stress episodes induced by acute ocean warming events. Under the current rate of warming, will corals be gone before the end of this century? Here we combine population genomics with biophysical and evolutionary modeling to investigate adaptive potential of a common reef-building coral from the Great Barrier Reef. To approach this task, we have developed a predictive model of polygenic adaptation in a system of multiple inter-connected populations that exist in a heterogeneous and changing environment. Applying this model to our coral species, we find that populations successfully adapt to diverse local temperatures along the range of the Great Barrier Reef despite high migrant exchange and should collectively harbor enough adaptive genetic variants to fuel region-wide thermal adaptation for another century and perhaps longer. In the same time, the model predicts that random thermal fluctuations will induce increasingly severe coral mortality episodes, which aligns well with observations over the last few decades.

## Introduction

Mass coral bleaching, caused by global warming, is devastating coral reefs around the world [1] but there is room for hope if corals can adapt to increasing temperatures from generation to generation [2]. Many coral species have wide distributions that span environments that differ dramatically in their thermal regimes, demonstrating that efficient thermal adaptation has occurred in the past [3]. But can coral adaptation keep up with the unprecedentedly rapid current rate of global warming [4]? One way for corals to achieve rapid thermal adaptation is through genetic rescue, involving the spread of existing heat tolerance alleles from warm-adapted populations to now-warming regions via larval migration [5,6]. We have previously demonstrated the presence of genetic variants conferring high thermal tolerance in a naturally warm low-latitude population of *A*. *millepora* on the Great Barrier Reef (GBR, [5]). It can be assumed that the effectiveness of GBR-wide adaptation based on these pre-existing variants would depend on the prevailing migrant exchange pathways and on total amount of genetic variation in populations of this coral. While considerable genetic connectivity along the latitudinal range of the GBR has been documented in corals [7–9], previous approaches have not been able to resolve the directionality of the migrant exchange to confirm that redistribution of heat-tolerance alleles from warm- to cooler-adapted populations is indeed taking place. In addition, recent declines in coral cover [10] could have already taken a toll on the total amount of standing genetic variation. Here, we used population genomics coupled with model-based allele frequency spectrum (AFS) analysis to establish directionality of migrant exchange and estimate contemporary and historical effective population sizes (measure of genetic diversity) in *Acropora millepora*, a common but heat sensitive reef-building coral representing the most ecologically prominent and diverse coral genus in the Indo-Pacific. The genomics-based migration rates were cross-validated with biophysical model of larval dispersal. The resulting demographic estimates were used to parameterize a newly developed metapopulation adaptation model to predict future persistence of *A*. *millepora* on the GBR.

## Results and discussion

### Locations and genotyping

**Fig 1:**
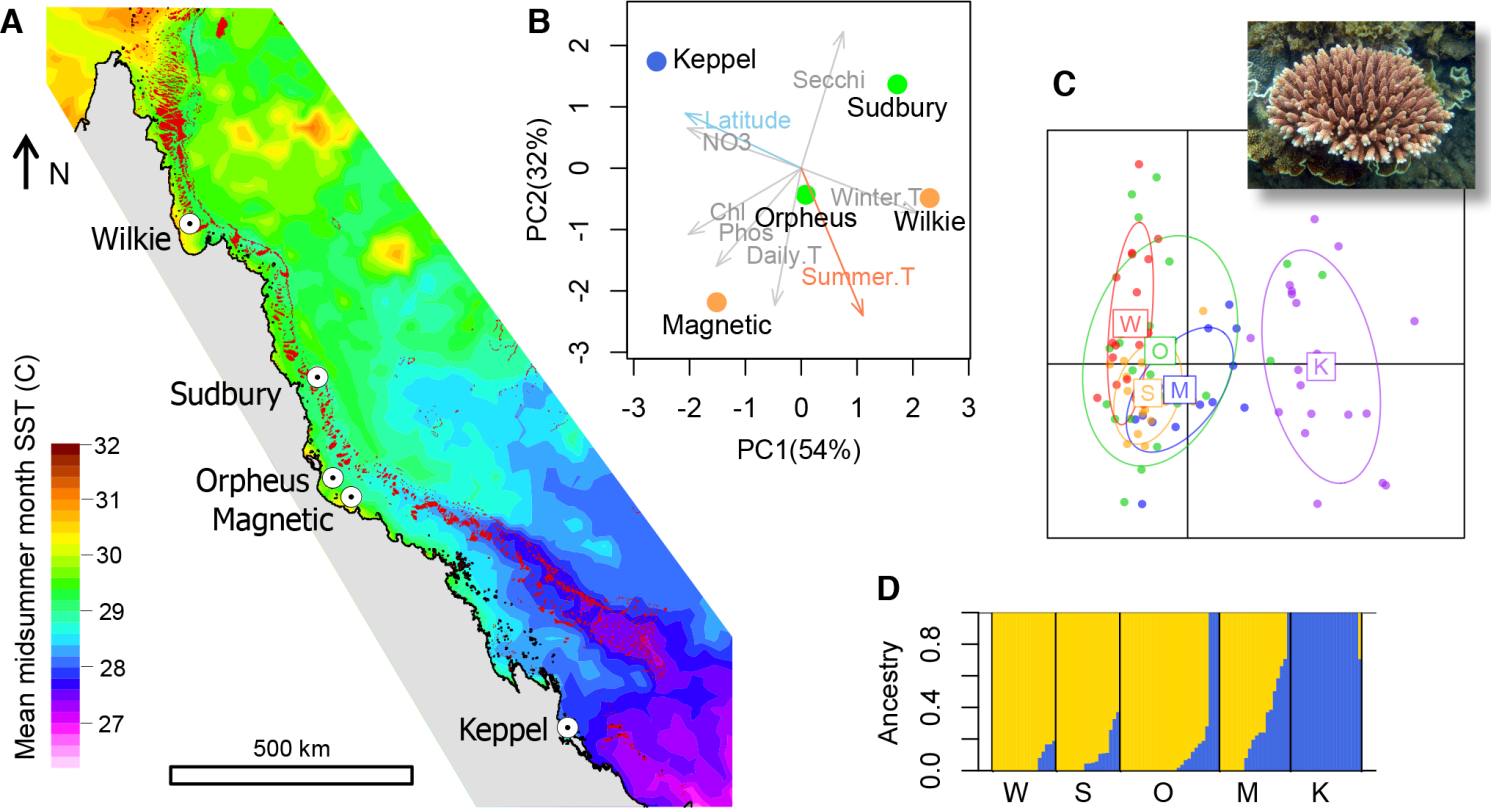
The population setting. (A) Locations of sampled populations where mean midsummer month sea surface temperature differed by up to ~3°C. (B) Principal component analysis of water quality and temperature parameters at the sampled locations. Winter.T—10% quantile of winter temperature, Summer.T– 90% quantile of summer temperature, Daily.T– 90% quantile of daily temperature range, Phos–total dissolved phosphorus, Chl–chlorophyll, NO3 –nitrate, Secchi–Secchi depth (water clarity). Locations are colored according to summer temperature as in panel A. (C) Principal component analysis of genome-wide genetic variation (inset–*Acropora millepora*). Centroid labels are initial letters of population names as in panel A. (D) ADMIXTURE plot of ancestry proportions with *K* = 2 (the lowest cross-validation error was observed with *K* = 1). Analyses on panels C and D were based on 11,426 SNPs spaced at least 2.5 kb apart and not including *F*_ST_ outliers.

We used samples collected in 2002–2009 from five populations of *A*. *millepora* along the latitudinal range of the GBR (Fig 1A). Environmental parameters (obtained from http://eatlas.org.au/) varied widely among these locations (Fig 1C). Importantly, maximum summer temperature (the major cause of bleaching-related mortality) followed the latitudinal gradient with one notable exception: one of the near-shore populations from the central GBR (Magnetic Island) experienced summers as hot as the lowest-latitude population examined here (Wilkie Island, Fig 1A and 1B).

We genotyped 18–28 individuals per population using 2bRAD [11] at >98% accuracy and with a >95% genotyping rate. Analysis of population structure based on ~11,500 biallelic SNPs agreed with previous results [8,12] and revealed very low levels of genetic divergence, with only the Keppel Islands population being potentially different from the others (Fig 1C and 1D). Pairwise *F*_ST_ did not exceed 0.014 even between the southernmost and northernmost populations (Keppel and Wilkie). We did not verify whether these *F*_ST_ measures were significant because our main statistical analysis was based AFS modeling.

### Demographic subdivision and migration patterns

Here we applied Diffusion Approximation for Demographic Inference (*∂a∂i*, [13]), a methodology of demographic analysis based on allele frequency spectrum (AFS). AFS is essentially a histogram of number of genetic variants binned by frequency (S1 Fig). AFS-based analysis is enabled by the multitude of molecular markers provided by next-generation sequencing and offers a number of advantages compared to classical population genetics approaches applied previously to GBR corals [7–9,14], Most importantly, AFS analysis does not rely on assumptions of genetic equilibrium (stability of population sizes and migration rates for thousands of generations) or equality of population sizes. It can fit any user-defined demographic model, for example involving asymmetric migration or population growth and declines, to maximize the likelihood of generating the observed AFS. This approach also allows likelihood-based model comparisons (likelihood ratio tests or tests based on Akaike Information Criterion, AIC) to prove the importance of parameters included in the model.

We used bootstrap-AIC approach to confirm that our populations are separate demographic units. For each pair of populations we generated 120 bootstrapped datasets by resampling genomic contigs and performed delta-AIC comparison of two demographic models, a split-with-migration model and a no-split model (S2 Fig). The split-with-migration model assumed two populations that split some time *T* in the past with potentially different sizes *N1* and *N2*, and exchange migrants at different rates (*m12* and *m21*) depending on direction. The no-split model allowed for ancestral population size to change but not for a population split, so the experimental data were modeled as two random samples from the same population of size *N*. The majority of bootstrap replicates (64–100%) showed AIC advantage of the split-with-migration model for all pairs of populations except Sudbury-Magnetic (41% support, S2 Fig). This indicates that most *A*. *millepora* populations on the GBR are in fact demographically distinct, despite often non-significant *F*_ST_ reported by previous studies based on allozymes [7,15] and microsatellite markers [8], or our own PCA and ADMIXTURE results (Fig 1C and 1D). This underscores the higher sensitivity of AIC-base AFS analysis compared to classical equilibrium population genetics methods.

**Fig 2:**
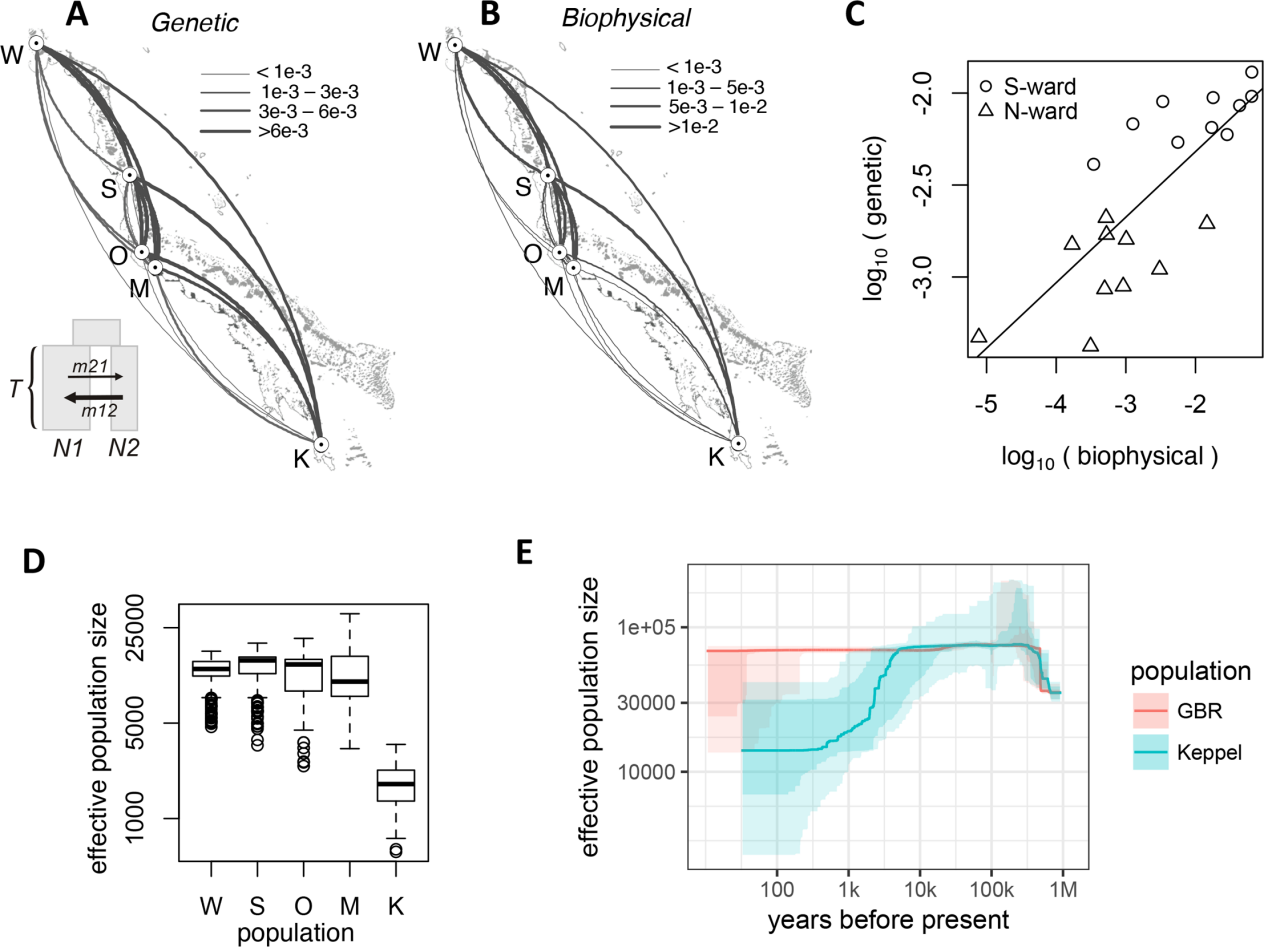
Estimated demography of *A*. *millepora* populations on the GBR. (A) Arc-plot of migration rates among populations reconstructed from population genomic data. Inset: *∂a∂i* model used: ancestral population splits into two populations of unequal sizes (N1 and N2) some time T in the past, these populations exchange migrants at different rates depending on direction. (B) Migration rates according to the biophysical model. On panels A and B, the arcs should be read clockwise to tell the direction of migration; line thickness is proportional to the migration rate. (C) Correlation between log-transformed biophysical and genetic migration rates (Mantel *r* = 0.58, *P* = 0.05). (D) Box plot of effective population sizes inferred by the split-with-migration model (panel A) across all population pairs and bootstrap replicates. (E) Historical effective population sizes inferred by *stairwayPlot* for the Keppel population and pooled Sudbury, Orpheus and Magnetic populations (GBR). The line is median of 200 bootstrap replicates, light shaded area is 95% credible interval, dark-shaded area is 75% credible interval.

AFS-based analysis allows statistically rigorous estimation of unidirectional migration rates between populations. The classical *F*_ST_−based approach only allows estimating bi-directional migration rate [15] and even this calculation has been criticized because its underlying assumptions are rarely realistic [16]. We determined unidirectional migration rates from the split-with-migration model and estimated their confidence limits from bootstrap replicates. In theory, migration rate can be confounded with population divergence time, since in the AFS higher migration often looks similar to more recent divergence [17]. To confirm that the model with ancient population divergence and migration is preferable to the model with very recent divergence and no migration, we performed the delta-AIC bootstrap comparison between these models and obtained overwhelming support for the model with ancient divergence and migration (S3 Fig). Notably, for all pairwise analyses migration in southward direction exceeded northward migration, and this difference was significant in seven out of ten pairwise comparisons (Fig 2A and S2A Fig). Linear mixed model analysis of direction dependent median migration rates with a random effect of destination (to account for variation in total immigration rate) confirmed the overall significance of this southward trend (*P*_MCMC_ <1e-4). Full listing of parameter estimates and their bootstrap-derived 95% confidence limits is given in S1 Table.

To investigate whether the southward migration bias was due to higher survival of warm-adapted migrants (due to ongoing sea surface temperature increase) rather than currents, we developed a biophysical model of coral larval dispersal on the Great Barrier Reef. This model quantified the per-generation migration potential among coral reef habitat patches in the GBR based on ocean currents and parameters of larval biology [18,19], the latter including pre-competency period, competency period and mortality rate [20]. The biophysical model predicted very similar migration rates as our genetic model (Mantel *r* = 0.58, *p* = 0.05), recapitulating the southward bias (Fig 2A–2C). Importantly, the same southward bias was predicted for population pairs in which southward migration corresponded to movement to the same-temperature or even to warmer location, such as migrations to the Magnetic Island. This indicates that southward migration bias is predominantly driven by ocean currents and not by preferential survival of warm-adapted coral genotypes migrating to cooler locations. More generally, this result indicates that currents remain the major factor affecting larval dispersal in our study species, with environmental selection pressures playing comparatively minor, if any, role.

### Genetic diversity trends

The GBR has warmed considerably since the end of last century [21], which may have already reduced genetic diversity in *A*. *millepora* populations. We used *∂a∂i* to infer effective population sizes, which is a measure of genetic diversity and one of the key parameters determining the population’s adaptive potential [22]. The results of the split-with-migration model (Fig 2A) were consistent for all population pairs and indicated that Keppel population was about one-fifth the size of others (Fig 2D and 2E). This result was not surprising since the Keppel population has frequently suffered high mortality due to environmental disturbances compared to the other populations studied [12]. To investigate whether there was a detectable recent drop in genetic diversity associated with GBR-wide coral decline [10] we have used *stairwayPlot*, a model-free method of past effective population size reconstruction based on AFS [23]. Reconstruction of the most recent changes draws information from the rarest alleles and therefore requires large sample sizes, which is why we pooled three highly similar populations from the Central GBR (Sudbury, Orpheus, and Magnetic) to increase the sample size to 84 individuals. We also analyzed Keppel population separately since it was distinct from the rest (Fig 1D and 1E) and was less genetically diverse (Fig 2D). This analysis confirmed the long-term decline of the Keppel population, possibly since the time of its separation from the main GBR stock, but did not find significant recent decline in the central GBR (Fig 2E). To see if a very recent decline was at all detectable with a sample size of 84, we used SLiM [24] to simulate evolution of 20,000 2bRAD loci in ten populations exponentially declining from 30,000 individuals to 1/10th or 1/3rd of that size over the last 20 generations (in our coral this would correspond to approximately 100 years). As a control, we simulated populations maintaining their size. With the sample size of 84 we could detect the ten-fold population crash in nine cases out of ten (albeit with low confidence) but we could not detect the three-fold decline (S4 Fig). Thus, although we did not detect recent drop in genetic diversity in our species, substantial population decline might still be occurring.

### Metapopulation adaptation model

To evaluate whether local thermal adaptation could facilitate rapid adaptation of the whole *A*. *millepora* metapopulation to the simulated gradual warming, we developed an individual-based polygenic model of metapopulation adaptation in the SLiM software environment [24]. The model’s code is highly flexible and can simulate any number of populations with any configuration of population sizes, migration rates, and environmental trends. The number and effect sizes of QTLs, mutation rate, heritability, and breadth of tolerance can also be varied in this model. Here, we used population sizes and migration rates inferred from the genetic analysis (Fig 2A and 2D) and incorporated differences in mid-summer monthly mean temperature among populations (Fig 1A). Initially, populations were allowed to adapt to local thermal conditions while exchanged migrants and were at the state of genetic equilibrium at the start of the warming periods (S5 Fig). Warming consists in the temperature increase at a rate of 0.05°C per generation in all populations, corresponding to the projected 0.1°C warming per decade [25]. Both during pre-adaptation and warming periods the temperature was allowed to fluctuate randomly between generations to approximate El Nino Southern Oscillation (ENSO) or similar but random acute temperature events. During the warming period, a population declining in fitness would shrink in size and stop contributing migrants to other populations.

### Adaptation based on standing genetic variation

**Fig 3:**
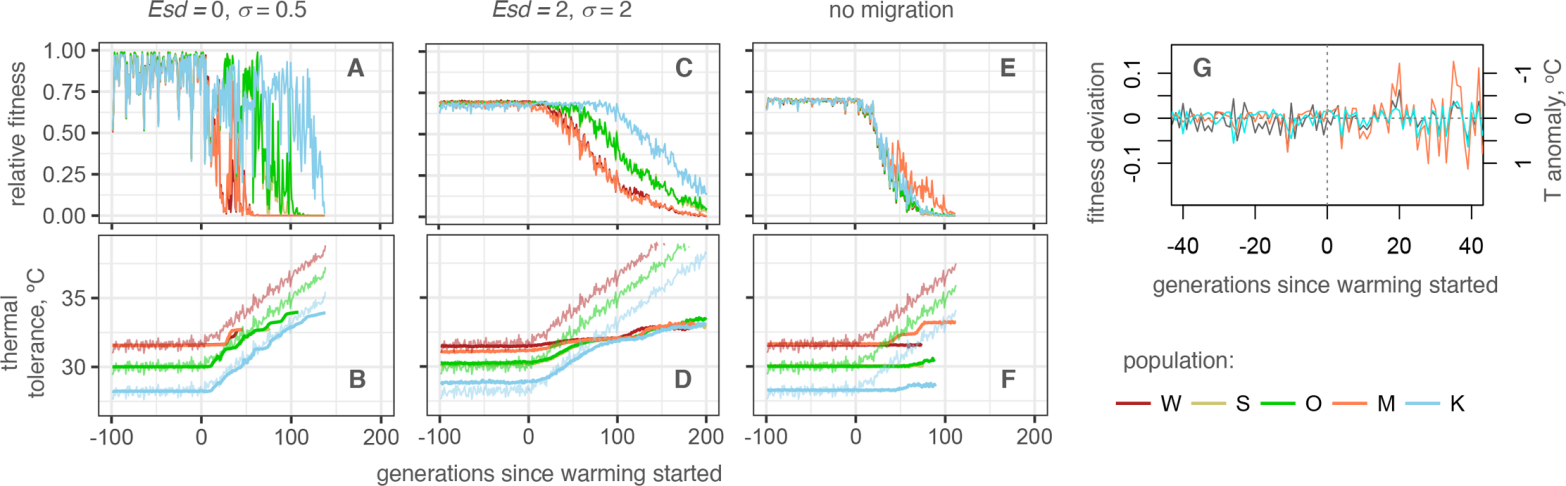
Modeling coral metapopulation persistence under warming. (A, C, E): Mean fitness, relative to maximum attainable with perfect heritability. (B, D, F): Mean phenotype (thick lines) and modeled temperatures (thin noisy lines). (A, B): Settings for the most efficient selection (perfect heritability, narrow tolerance). (C, D): Settings for the least efficient selection (low heritability, broad tolerance). (E, F): Intermediate heritability and tolerance settings (*Esd* = 1, *σ* = 1) with no migration. Warm-adapted populations (W and M) are shown as red-tint traces, populations from mild thermal regime (S and O) are green-tint traces, and the cool-adapted population (K) are the blue traces. Note close similarity between traces for pairs of populations pre-adapted to the same temperature (W, M and S, O). (G) Sensitivity of populations to random thermal anomalies increases under warming. Modeled temperature anomalies are shown as grey line, fluctuations in populations’ fitness–as colored lines (residuals from loess regression over fitness traces at *Esd* = 1, *σ* = 1; Wilkie: orange line, Keppel: blue line). The sign of temperature anomalies is inverted to better reveal the correspondence between rise in temperature and drop in fitness. Mutation rate was 1e-6 per locus per gamete in all simulations shown.

The fist important result of the model was that high migration rates (on the order of 0.1–1% of immigrants every generation, Fig 2C and S1 Table) did not lead to “migrational meltdown” [26] of local adaptation: all populations successfully adapted to local thermal conditions, although at the settings for lower efficiency of selection (low heritability and/or broad tolerance) this adaptation became increasingly imperfect (Fig 3B and 3D and S7 Fig). Moreover, under all parameter settings the pre-adapted metapopulation as a whole was able to persist through the gradual warming for at least 20–50 generations (100–250 years) although the initially warm-adapted populations were going extinct relatively rapidly (Figs 3, S6 and S7). Migration substantially contributed to this persistence (Fig 3E and 3F), underscoring the importance of divergent local adaptation and genetic rescue [5,6] in promoting and redistributing the standing genetic variation.

A notable tendency observed with all parameter settings was that during warming the fitness (and hence the size) of adapting populations began to fluctuate following random thermal anomalies, and the amplitude of these fitness fluctuations increased as the warming progressed even though the amplitude of thermal anomalies did not change (Fig 3G). These fluctuations correspond to severe mortality events induced by thermal extremes that can occur as a result of ENSO and heat waves and affected warm-adapted populations most, which very much resembles the situation currently observed throughout the world [1].

### Adaptation proceeds despite inefficient selection

Efficiency of selection depends on how strongly the phenotype is determined by genotype (heritability), and also on how steeply the fitness declines if the phenotype does not match the environment (breadth of thermal tolerance). In our model, heritability becomes lower with more random variation (determined by the *Esd* parameter, Figs 3, S6 and S7) added to the breeding value. Lower heritability notably diminished the efficiency of local adaptation–during pre-adaptation period, mean fitness of each population was lower (S6 Fig) and the population’s mean phenotype failed to achieve full match to the environment (S7 Fig), ostensibly due to less efficient selection against maladapted immigrants. Yet, lower heritability did not result in reduced persistence of the metapopulation (Figs 3, S5 and S6). This is good news for reef-building corals since heritability of thermal tolerance in them is expected to be low: much of natural variation in this trait is due to the type of algal symbionts (*Symbiodinium* spp. [27]). Photo-symbionts are not transmitted from parent to offspring in the majority of coral species [28], and although host genetics can have some effect on the choice of *Symbiodinium* in the next generation [29] environment has stronger effect on this association [27,30].

Broader thermal tolerance (determined by parameter σ, Figs 3, S6 and S7) also reduces the efficiency of selection but increases the population’s mean fitness, counteracting the fitness-diminishing effect of lower heritability (S6 Fig). During warming, it prevents extinction despite increasingly poorer match between the population’s mean phenotype and the environment, and thus facilitates longer persistence (Figs 3, S6 and S7). It is also notable that both low heritability and broader tolerance decrease the sensitivity of populations to random thermal fluctuations (Fig 3A and 3C and S6 Fig).

### Model uncertainties

There are many uncertainties in our model associated with coral biology. Below we argue that, while more research is certainly needed to resolve them, our parameter settings were for the most part set to under-estimate adaptive potential.

**Fig 4:**
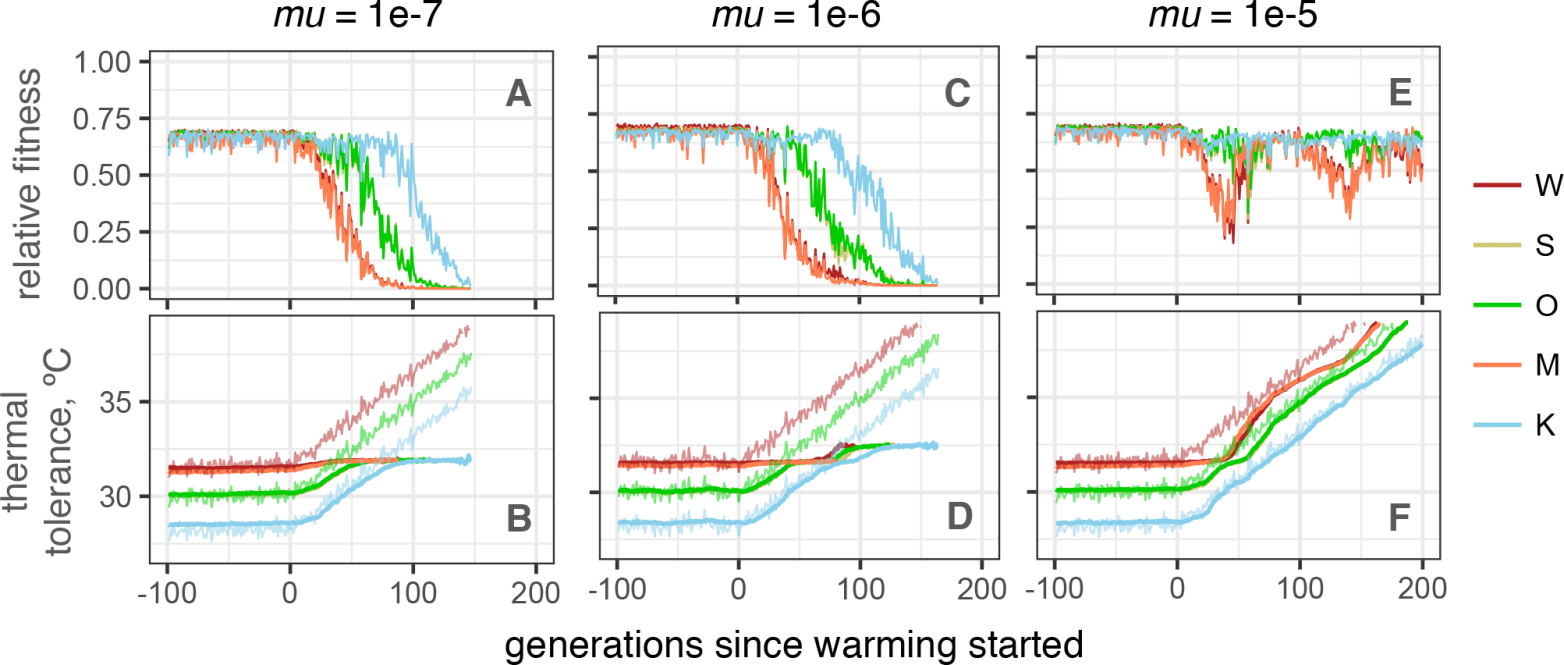
Effect of mutation rate on population persistence. (A, C, E): Mean fitness, relative to maximum attainable with perfect heritability. (B, D, F): Mean phenotype (thick lines) and modeled temperatures (thin noisy lines). Mutation rate (*mu*) per locus per gamete is listed above the graphs; effect sizes of new mutations were drawn from a normal distribution with mean 0 and standard deviation 0.2°C. Adaptation to local thermal conditions and initial adaptive response based on genetic rescue happen efficiently even under low mutation rate (1e-7), but further evolution is only possible at high mutation rate (1e-5). All simulations shown share intermediate selection efficiency settings: *Esd* = 1, *σ* = 1.

Mutation rates are generally difficult to estimate [31] and therefore in our model we had to rely on order-of-magnitude guesses. Encouragingly, even under relatively low per-locus mutation rate of 1e-6 per gamete per generation [32] we have observed occasional “evolutionary rescue” events: brief periods of accelerated phenotypic evolution due spread of novel beneficial mutations [33]. These events substantially contributed to the metapopulation persistence (Fig 3B and S7 Fig). Furthermore, even under ten-fold lower mutation rate (1e-7) the initial adaptive response during the first ~100 generations was still observed (Fig 4A), although there was no subsequent adaptation. While ten-fold higher mutation rate (1e-5) allowed for indefinite adaptation (Fig 4C), such a high mutation rate is unlikely to be realistic [32,34].

**Fig 5:**
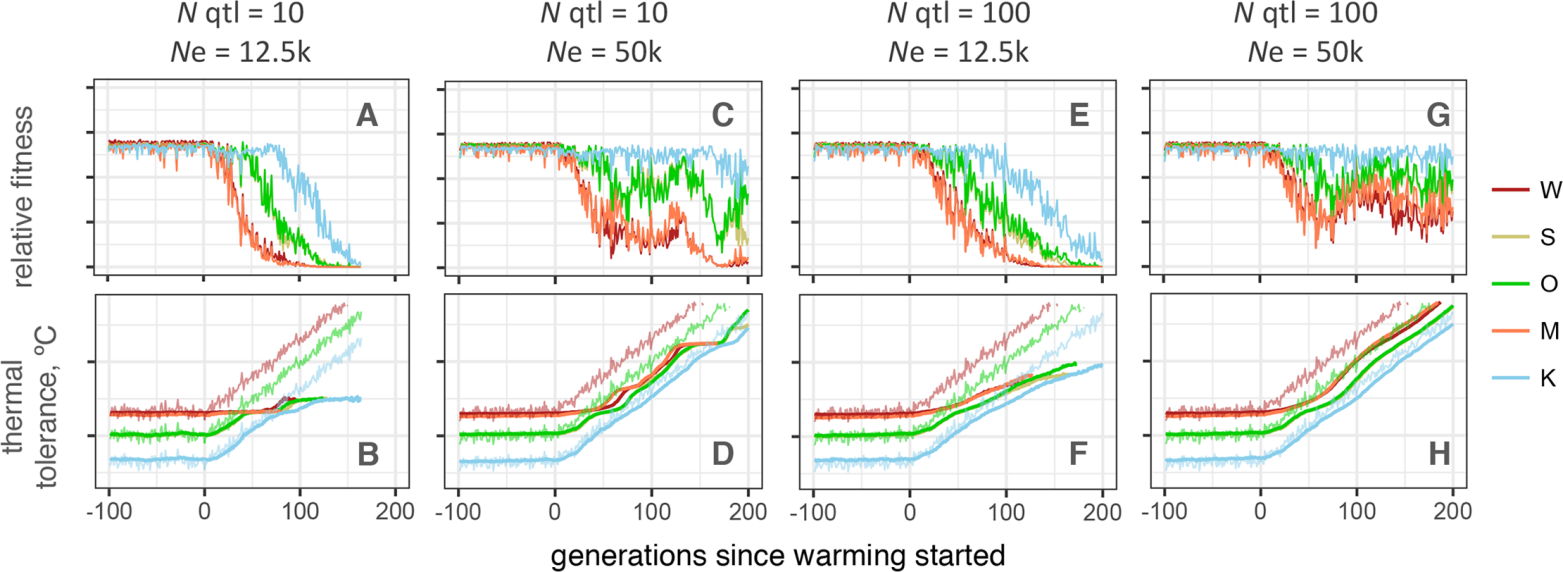
Larger population size and finer genetic architecture facilitate population persistence under warming. (A, C, E, G): Mean fitness, relative to maximum attainable with perfect heritability. (B, D, F, H): Mean phenotype (thick lines) and modeled temperatures (thin noisy lines). Number of QTLs (*N* qtl) and population sizes (*N*e) are listed above graphs (K population size is five-fold smaller in all cases). With 100 QTLs, their effect sizes are proportionally smaller to enable the same total genetic variance as with 10 QTLs. Both larger population size (C, D) and finer genetic architecture (E, F) improve population persistence, and combination of the two might enable populations to adapt indefinitely (G, H). All simulations shown share intermediate selection efficiency settings: *Esd* = 1, *σ* = 1, and *mu* = 1e-6.

Changes in two other parameters could result in considerably longer persistence: larger population sizes and more fine-grained genetic architecture (more QTLs with less effect). Both of these strongly facilitate adaptation beyond the initial “genetic rescue” period (Fig 5). Unlike the high mutation rate, these settings are relatively realistic. Coral population size is likely larger than assumed in our model, which used effective population sizes suggested by genetic analysis as census sizes. However, in highly fecund marine organisms effective population sizes tend to be much smaller than census sizes, sometimes by orders of magnitude [35]. It is also notable that *stairwayPlot* predicted substantially larger effective population sizes than the *∂a∂i*-derived estimates used for the model (Fig 2D and 2E). As for genetic architecture, our assumption of only ten thermal QTLs was conservative; the actual number of thermal QTLs in acroporid corals is likely much larger [36]. However, there is currently no data on the distribution of effect sizes of these QTLs, which would be an important subject for future research to improve the model.

As for breadth of thermal tolerance, in simulations shown on Fig 3 σ = 0.5 and σ = 2 corresponded to 86% and 13% decline in fitness if the individual’s phenotype mismatched the environment by 1°C. The existing data on the breadth of coral thermal tolerance are somewhat conflicting. One study shows that acroporid corals can successfully acclimatize to environments differing in maximum temperatures by as much as 2°C [37]; however, another study found that coral grew 52–80% more slowly when transplanted among locations differing by 1.5°C average temperature, [38]. Although it is not possible to directly interpret these results in terms of breadth of thermal tolerance function, the former study likely supports the broader tolerance setting while the latter study suggests narrower tolerance. It must also be noted that both these studies involved *in situ* transplantations and hence the effect of temperature remains confounded with colony history and other local fitness-affecting environmental parameters. Also, adult corals likely have narrower tolerance than larvae and recruits, which are expected to exhibit non-reversible developmental plasticity associated with metamorphosis and establishment within a novel environment [39]. One particularly important event during this developmental transition is establishment of association with local algal symbionts. Since symbionts also adapt to local thermal conditions [30] this would elevate the fitness of the coral host despite possible mismatch between its own genetically determined thermal optimum and local temperature, which we can model as broadening of the thermal tolerance. Future experiments that expose multiple genetically distinct coral individuals to a range of temperatures under controlled laboratory settings are required to rigorously quantify variation in thermal tolerance curves in natural populations.

It could be argued that our samples are genetically out of date, not capturing the effects of recent disturbances such as mass bleaching, large cyclones and a Crown-of-Thorns outbreak that have happened on the GBR since the time of their collection (2002–2009). However, very recent demographic events (in this case, 2–3 generations ago) are undetectable at the level of neutral genetic variation (S4 Fig) unless the study’s sample size is comparable to the number of disturbance-surviving individuals (i.e., either when the disturbance was truly catastrophic or the sample size is very large). Thus, our samples can still be considered representative of major patterns of genetic diversity of our study species.

Finally, our model assumed that recovery from high mortality events would happen without impediment, through reseeding by survivors and migrant influx from other coral populations. However, severe mortality across large spatial scales or ecological feedbacks such as shifts to an alternative ecological stable state [40] might substantially decrease the rate of reseeding and recovery of affected reefs. In that case, the increase in severity of bleaching-related mortality might lead to much faster coral extinction than predicted by our model.

### Implications for reef management

We found that genetic diversity of *Acropora millepora* was not yet strongly affected by climate change and that the migration patterns were well positioned to facilitate persistence of the GBR metapopulation for a century or more. Our results underscore the pivotal role of standing genetic variation and migrant exchange in the future metapopulation persistence, suggesting management interventions such as assisted gene flow [41] by moving adult reproductively active colonies or by outplanting lab-reared offspring produced by crossing corals from different populations. With the estimated natural migration rates on the order of 0.1–1% (10–100) migrants per generation, human-assisted genotype exchange could appreciably contribute to the genetic rescue without risking disruption of the natural local adaptation patterns [42]. What might get in the way of assisted gene flow is adaptation of transplanted corals to other environmental parameters at home, for example, light levels or concentration of inorganic nutrients. The extent to which such adaptation can limit survival of transplanted corals and naturally dispersing larvae (the effect called “isolation by environment” [43] or “phenotype-environment mismatch” [44]) requires further study. Importantly, despite good prospects for short-term adaptation, coral populations are predicted to become increasingly more sensitive to random thermal anomalies, especially in the originally warm-adapted populations. The 10–85% mortality in the Northern GBR as a result of 2016 bleaching event [45] could be a particularly sobering recent manifestation of this trend. Finally, to validate the model’s predictions and further fine-tune its parameters, long-term monitoring of genetic variation in natural coral populations must be initiated to track ongoing evolutionary changes.

## Materials and methods

### Ethics statement

This study relied predominantly on samples described by van Oppen et al [8] with addition of several samples from Orpheus and Keppel islands that were used in the reciprocal transplantation experiment described by Dixon et al [46]. These samples were collected under Great Barrier Reef Marine Park Authority permits number G99/441 and G09/29894.1.

### Genotyping

The samples were genotyped using 2bRAD [11] modified for Illumina sequencing platform; the latest laboratory and bioinformatics protocols are available at https://github.com/z0on/2bRAD_denovo. BcgI restriction enzyme was used and the samples retained for this analysis had 2.3–20.2 (median: 7.45) million reads after trimming and quality filtering (no duplicate removal was yet implemented in this 2bRAD version). The reads were mapped to the genome of the outgroup species, *Acropora digitifera* [47,48], to polarize the allelic states into ancestral (as in *A*. *digitifera*) and derived, e.g., [49,50]. Genotypes were called using GATK pipeline [51].

Preliminary analysis of sample relatedness using vcftools [52] revealed that our samples included several clones: four repeats of the same genotype from the Keppel Island (van Oppen et al [8] samples K210, K212, K213 and K216), another duplicated genotype from Keppel (samples K211 and K219), and one duplicated genotype from Magnetic Island (samples M16 and M17). All other samples were unrelated. We took advantage of these clonal replicates to extract SNPs that were genotyped with 100% reproducibility across replicates and, in addition, appeared as heterozygotes in at least two replicate pairs (script replicatesMatch.pl with hetPairs = 2 option). These 7,904 SNPs were used as “true” SNP dataset to train the error model to recalibrate variant quality scores at the last stage of the GATK pipeline. During recalibration, we used the transition-transversion (Ts/Tv) ratio of 1.438 determined from the “true” SNPs to assess the number of false positives at each filtering threshold (as it is expected that an increase of false positive calls would decrease the Ts/Tv ratio towards unity). We chose the 95% tranche, with novel Ts/Tv = 1.451. After quality filtering that restricted the calls to only bi-allelic polymorphic sites, retained only loci genotyped in 95% or more of all individuals, and removed loci with the fraction of heterozygotes exceeding 0.6 (possible lumped paralogs), we ended up with 25,359 SNPs. In total, 2bRAD tags interrogated 0.18% of the genome. The genotyping accuracy was assessed based on the match between genotyped replicates using script repMatchStats.pl. Overall agreement between replicates was 98.7% or better with the heterozygote discovery rate (the fraction of matching heterozygote calls among replicates) exceeding 96%. All but one representative of each clonal group were excluded from all subsequent analysis.

### Genome-wide genetic divergence

To begin to characterize genome-wide divergence between populations we used pairwise genome-wide Weir and Cockerham’s *F*_ST_ calculated by vcftools [52], principal component analysis (PCA) using R package adegenet [53], and ADMIXTURE [54]. For PCA and ADMIXTURE, the data were thinned to keep SNPs separated by 5kb on average and by at least 2.5 kb, choosing SNPs with highest minor allele frequency (script thinner.pl with options ‘interval = 5000 criterion = maxAF’), resulting in 11,426 unlinked SNPs. The optimal K in ADMIXTURE analysis was determined based on the cross-validation procedure incorporated within ADMIXTURE software; the lowest standard error in cross-validation was observed at K = 1.

### Demographic analysis and bootstrapping

Prior to demographic analysis, Bayescan [55] was used to identify sites potentially under selection among populations, and 73 sites with q-value <0.5 were removed. This aggressive removal of potential non-neutral sites resulted in better agreement between bootstrap replicates compared to an earlier analysis where only 13 sites with q-value < 0.05 were removed. Demographic models were fitted to 120 bootstrapped datasets, which were generated in two stages. First, three alternatively thinned datasets were generated for which SNPs were randomly drawn to be on average 5 kb apart and not closer than 2.5 kb (10,042–10,074 SNPs in each). This time the SNPs were drawn at random to avoid distorting the allele frequency spectrum (unlike thinning for PCA and ADMIXTURE where the highest minor allele frequency SNPs were selected). Then, 40 bootstrapped replicates were generated for each thinned dataset by resampling contigs of the reference genome with replacement (script dadiBoot.pl). The fitted model parameters were summarized after excluding bootstrap replicates that fell into the lowest 15% likelihood quantile and the ones where model fitting failed to converge, leading to some parameters being undetermined or at infinity (less than 10% of total number of runs). Delta-AIC values were calculated for each bootstrap replicate that passed these criteria for both compared models, and summarized to obtain bootstrap support value, the percentage of replicates favoring the alternative model. While fitting *∂a∂i* models, the data for each population were projected to sample sizes maximizing the number of segregating sites in the analysis, resulting in 6143–7193 segregating sites per population. Initially, our models included a parameter designed to account for ancestral state misidentification rate when constructing the polarized AFS (e.g., [56]), but since this parameter was consistently estimated to be on the order of 0.001 and had negligible effect on the models’ likelihood, we removed it from the final set of models.

### Unit conversion

To convert *∂a∂i* -reported parameter values (θ, *T* and *M*) into time in years (*t*), effective population sizes in number of individuals (*Ne*) and migration rates (the fraction of the total population that are new immigrants in each generation, *m*), we estimated the mutation rate (μ) from the time-resolved phylogeny of *Acropora* genus based on the *paxC* intron [57], at 4e-9 per base per year. Although *A*. *millepora* can reproduce after 3 years [58] we assumed a generation time of 5 years reasoning that it would better reflect the attainment of full reproductive potential as the colony grows. Assuming a genome size of 5e+8 bases [47] the number of new mutations per genome per generation is 10. Since the fraction of the genome that is sequenced using 2bRAD was 1.8e-3 (calculated by dividing the total length of genotyped RAD loci by the size of the reference genome), the mutation rate per 2bRAD-sequenced genome fraction per generation is μ = 0.018. This value was used to obtain:

- Ancestral effective population size: *Ne* = *θ/*4*μ*
- Migration rate: *m* = *M* / 2*Ne*
- Time in years: *t* = 2*TNe •* 5

### Biophysical model

A spatially-explicit biophysical modeling framework [18,59] was used to quantify migration between coral reef habitats of the broader region surrounding the Great Barrier Reef, thereby revealing the location, strength, and structure of a species' potential population connectivity. The model’s spatial resolution of ca. 8 km coincides with hydrodynamic data for the broader region (1/12.5 degree; HYCOM+NCODA Reanalysis and Analysis product; hycom.org). Our biophysical dispersal model relies on geographic data describing the seascape environment and biological parameters capturing coral-specific life-histories. Coral reef habitat data are available from the UNEP World Conservation Monitoring Centre (UNEP-WCMC; http://data.unep-wcmc.org/datasets/1) representing a globally-consistent and up-to-date representation of coral reef habitat. To capture specific inter-annual variability, two decades of hydrodynamic data were used from 1992 to 2013 [60].

Coral-specific biological parameters for *A*. *millepora* included relative adult density (dependent on the habitat), reproductive output, larval spawning time and periodicity (e.g., the majority of colonies at Magnetic Island spawn a month earlier than the majority of colonies on other GBR sites [61]), maximum dispersal duration, pre-competency and competency periods, and larval mortality [20]. The spatially explicit dispersal simulations model the dispersal kernel (2-D surface) as a ‘cloud’ of larvae, allowing it to be concentrated and/or dispersed as defined by the biophysical parameters. An advection transport algorithm is used for moving larvae within the flow fields [62].

Simulations were carried out by releasing a cloud of larvae into the model seascape at all individual coral reef habitat patches and allowing the larvae to be transported by the currents. Ocean current velocities, turbulent diffusion, and larval behavior move the larvae through the seascape at each time-step. Larval competency, behavior, density, and mortality determine when and what proportion of larvae settle in habitat cells at each time step. When larvae encounter habitat, the concentration of larvae settling with the habitat is recorded at that time-step. From the dispersal data, we derived the coral migration matrix representing the proportion of settlers to each destination patch that came from a source patch, which is analogous to the source distribution matrix [63] and is equivalent to migration matrices derived from population genetic analysis. It is important to note that migration matrices extracted for the field sites represent the potential migration through all possible stepping-stones.

### Metapopulation adaptation model

The model was implemented in SLiM [24], the forward evolutionary simulator, by modifying the provided recipe “Quantitative genetics and phenotypically-based fitness”. The model simulated Wright-Fisher populations with discreet generations. At the start of the simulation, populations were established with specified pairwise migration rates. Mutations (at the rate of 1e-6 per locus per gamete) had the effect size drawn from a normal distribution with mean zero and specified standard deviation. To rapidly generate and equilibrate the standing genetic variation, we used the fact that the allele frequency spectrum is the function of the product of population size and mutation rate. Since smaller populations equilibrate proportionally faster and are much faster to simulate, the first 5,000 generations were performed at 100-fold smaller population size than the final target value (12,500 for all populations except K, which was five times smaller) but 100-fold higher mutation rate, followed by 5,000 generations with 10-fold smaller population size and 10-fold higher mutation rate, followed by 10,000 generations with the target population size and mutation rate. This step-wise strategy resulted in rapid generation and equilibration of genetic diversity both within individual populations and in the whole metapopulation (S5 Fig). The phenotype of each individual was calculated as breeding value (sum of all QTL effects) plus Gaussian-distributed noise (of the magnitude set by the *Esd* parameter) to simulate a non-heritable phenotypic component. Then, fitness of each individual was calculated based on the difference between the individual’s phenotype (thermal optimum), temperature of the environment, and the setting for the breadth of thermal tolerance curve (σ parameter, the standard deviation of the Gaussian slope of fitness decline away from the phenotypic optimum). Each generation parents were chosen to produce the next generation according to their fitness; parents for immigrant individuals were chosen from among individuals in the source population. New mutations at QTLs happened at the specified rate at the transition to the next generation and the effect of a new mutation was added to the previous QTL effect. To model fitness-dependent population demography, we implemented linear scaling of the population size and migration rates with the population’s mean fitness. In the model described here this scaling was applied during 500 generation preceding warming and during the warming period, so that populations declining in fitness relative to their historical level shrunk in size and stopped contributing migrants to other populations. Genetic variation shown on S5 Fig was calculated as standard deviation of breeding values, representing the average difference between genetically-determined thermal tolerance of an individual and the population’s mean in °C (genetic variance is this value squared).

Adjustable model parameters and their settings in this study were:

- Number of QTLs and the distribution of their effect sizes. To keep the model conservative, we modeled only ten QTLs with normal distribution of effect sizes with a standard deviation of 0.2°C. To model the effect of more dispersed genetic architecture while keeping the total genetic variation the same, we modeled 100 QTLs with mean effect size of 0.063 (since genetic variance is proportional to the square of QTL effect size). We have confirmed that these settings resulted in the same level of total genetic variation as ten QTLs with mean effect size of 0.2.
- Dominance of QTLs (set to 0.5 in our simulation).
- Breadth of thermal tolerance: standard deviation of the Gaussian curve describing decline in fitness away from phenotypic optimum. We modeled three tolerance settings, 0.5, 1 and 2, which corresponded to 86%, 40% and 13% fitness drop when the environment mismatched phenotypic optimum by 1°C.
- Non-heritable phenotypic component: standard deviation of a normal distribution with mean zero from which a random value is drawn to be added to the sum of QTL effects when computing phenotype. Setting this parameter to zero corresponds to trait heritability of one. Higher values of this parameter imply heritability less than one; however, the exact value of heritability (the proportion of phenotypic variation explained by genetics) could vary depending on the amount of genetic variation.
- Mutation rate. It was set to 1e-6 per locus per gamete for the majority of runs, with the exception of runs exploring lower (1e-7) or higher (1e-5) ends of theoretically realistic per-locus mutation rates [32].

The model’s code is designed for general modeling of polygenic adaptation in metapopulations. It can read user-supplied files of environmental conditions, population sizes and migration matrices for an arbitrary number of populations.

Here, we modeled our five populations with effective population sizes and pairwise migration rates inferred by *∂a∂i*. We modeled identical thermal trends across populations with population-specific offsets. During the pre-adaptation period lasting 20,000 generations, the temperature was constant on average but experienced random fluctuations across generations drawn from a normal distribution with a standard deviation of 0.25°C (to approximate ENSO events). The temperature was offset by +1.6°C in Wilkie and Magnetic populations and by -1.8°C in the Keppel population, to model differences in midsummer monthly mean temperature among populations (Fig 1). After 20,000 generations, a linear increase at 0.05°C per generation was added to simulate warming.

All combinations of parameter settings were run ten times to ensure consistency. We found that with population sizes in thousands, such as in our case, the results were very consistent among independent runs. We therefore did not aggregate results over many replicated runs but instead show one randomly chosen run for each tested parameter combination.

## Supporting information

S1 Table Summary of demographic analysis based on split-with-migration model (Fig 2A). “lo” and “hi” values are bootstrap-based 95% confidence limits.
(PDF)

S1 Fig Example of two-population *∂a∂i* model fit. (A) The model: ancestral population splits into two populations of unequal sizes (N1 and N2) some time T in the past, which exchange migrants with different rates depending on direction. (B) Observed allele frequency spectrum comparing Wilkie (W) and Keppel (K) populations. (C) Allele frequency spectrum generated by the fitted model. (D, E) Map and histogram of residuals (absolute scale).
(PDF)

S2 Fig Bootstrap analysis of migration rates, divergence times, and population subdivision. (A) Migration among population pairs, with bootstrap-derived 95% confidence intervals. The pairs are identified on the x-axis and sorted by increasing geographical distance. Black bars–southward migration, grey bars–northward migration. (B) Boxplot of divergence times (in years, y-axis) between pairs of populations (x-axis) across bootstrap replicates. (C) Models being compared: the split model (left) implies populations’ split into two different sizes (N1 and N2) at time T in the past, since when they exchanged migrants at unequal rates depending on direction. No-split model (right) allows for population size change at time T in the past but does not include population split: the two genotyped groups (p1 and p2) are regarded as two samples from the same population. (D-M) Histograms of delta-AIC values comparing split and no-split models (panel A) for bootstrap replicates (bootstrap was performed over genomic contigs of the draft genome of *A*. *digitifera*). Positive numbers indicate support for the split model. The letters on top of each panel identify compared populations, the number is the proportion of positive bootstrap replicates (i.e., bootstrap support for the full model).
(PDF)

S3 Fig Delta-AIC bootstrap comparison of models with and without migration (A), to confirm that the model with migration and ancient divergence is preferable to the model with no migration but very recent divergence. (B-K) Histograms of delta-AIC values for bootstrap replicates comparing models with and without migration. Positive numbers indicate support for the model with migration. The letters on top of each panel identify compared populations, the number is the proportion of bootstrap replicates supporting the model with migration and ancient split. For all pairs of populations the model of ancient split with migration is strongly supported. (L, M) Example of residuals from the two models. Model without migration under-estimates the number of shared low-frequency polymorphisms and over-estimates the number of shared high-frequency polymorphisms.
(PDF)

S4 Fig Detection of exponential population decline in the last 100 years (20 generations) when sampling 84 individuals. For each population size change scenario, ten datasets (evolution of 20,000 2bRAD loci in a population of 30,000 individuals) were simulated in SLiM. The line is median of 200 *stairwayPlot* bootstrap replicates for each of these datasets, light shaded area is 95% credible interval, dark-shaded area is 75% credible interval. Despite generally high uncertainty about recent population sizes, tenfold decline (left column) is detectable as a drop in the median in 9 out of 10 cases, although rarely at the 95% confidence level. Threefold decline scenario (middle column) does not show a pattern different from the no-change scenario (right column).
(PDF)

S5 Fig Genetic variation within populations (colored lines) and in the whole metapopulation (dashed grey line) depending on heritability (*Esd*; lower *Esd* implies higher heritability) and breadth of thermal tolerance (*σ*). Total metapopulation genetic variation stays constant over the 500 generations preceding warming (up to generation 0), indicating genetic equilibrium. Note that at the settings for inefficient selection (high *Esd* and *σ*, panel H, F, and I) the variation within populations increases, especially at locations receiving many immigrants (all except W). In all cases, warming results in the rapid initial drop in total genetic variation. Genetic variation was calculated as standard deviation of breeding values across individuals.
(PDF)

S6 Fig Effect of broader thermal tolerance (σ, standard deviation of the Gaussian slope of fitness decline away from the phenotypic optimum, in °C) and lower heritability (i.e. higher *Esd*, standard deviation of normally distributed random value added to the breeding value when calculating individual’s phenotype) on mean population fitness (relative to the maximum attainable with perfect heritability). Rows of panels (A-C, D-F, G-I) correspond to different *σ* indicated to the right of the rows; columns of panels correspond to the same *Esd* value indicated above the column. Lower heritability strongly diminishes the fitness attained during pre-adaptation to local conditions, but mitigates the influence of random thermal anomalies. Broader tolerance also mitigates the effect of anomalies, partially rescues the drop in fitness due to low heritability, and promotes longer population persistence.
(PDF)

S7 Fig Effect of broader thermal tolerance (*σ*, standard deviation of the Gaussian slope of fitness decline away from the phenotypic optimum, in °C) and lower heritability (i.e. higher *Esd*, standard deviation of normally distributed random value added to the breeding value when calculating individual’s phenotype) on the mean thermal optima in populations (thick lines). Rows of panels (A-C, D-F, G-I) correspond to different *σ* indicated to the right of the rows; columns of panels correspond to the same *Esd* value indicated above the column. Thin noisy lines show temperature in the environment. Lower heritability diminishes the efficiency of local adaptation and directed evolution without affecting long-term persistence. Broader thermal tolerance has the same effect on local adaptation and directed evolution but improves population persistence.
(PDF)
