# Supplementary material for: Potential and limits for rapid genetic adaptation to warming in a Great Barrier Reef coral
Source: PLoS Genet. 2018 Apr 19;14(4):e1007220. doi: 10.1371/journal.pgen.1007220 (PMC5908067; doi:10.1371/journal.pgen.1007220)
Supplement: S1 Table — “lo” and “hi” values are bootstrap-based 95% confidence limits. (PDF) [file pgen.1007220.s001.pdf]

**pop1**	**pop2**	**nu1.median**	nu1.hi	nu1.lo	**nu2.median**	nu2.hi	nu2.lo	**T.median**	T.lo	T.hi	**m12.median**	m12.lo	m12.hi	**m21.median**	m21.lo	m21.hi
**W**	**S**	**9800**	5000	14000	**8000**	4200	14000	**290000**	110000	600000	**0.0020**	0.0008	0.0120	**0.0060**	0.0010	0.0130
**W**	**O**	**12000**	9600	14000	**7600**	4500	9900	**300000**	130000	700000	**0.0017**	0.0012	0.0021	**0.0094**	0.0061	0.0140
**W**	**M**	**11000**	7700	14000	**8000**	5500	12000	**220000**	110000	400000	**0.0015**	0.0009	0.0026	**0.0065**	0.0028	0.0110
**W**	**K**	**14000**	13000	16000	**1900**	1100	3100	**130000**	96000	180000	**0.0005**	0.0000	0.0008	**0.0054**	0.0031	0.0099
**S**	**O**	**13000**	8000	15000	**11000**	7400	14000	**170000**	96000	850000	**0.0011**	0.0003	0.0045	**0.0096**	0.0026	0.0150
**S**	**M**	**14000**	8800	15000	**13000**	7300	29000	**140000**	61000	720000	**0.0009**	0.0003	0.0025	**0.0130**	0.0053	0.0180
**S**	**K**	**15000**	13000	18000	**1500**	840	2700	**150000**	110000	300000	**0.0004**	0.0000	0.0018	**0.0086**	0.0043	0.0150
**O**	**M**	**11000**	2700	14000	**11000**	4900	16000	**140000**	60000	580000	**0.0016**	0.0008	0.0170	**0.0090**	0.0007	0.0130
**O**	**K**	**15000**	13000	19000	**1800**	750	2600	**140000**	94000	320000	**0.0009**	0.0005	0.0019	**0.0068**	0.0041	0.0170
**M**	**K**	**18000**	15000	21000	**2800**	830	3400	**370000**	120000	430000	**0.0021**	0.0012	0.0024	**0.0041**	0.0033	0.0170
