# Supplementary figures and images for: Potential and limits for rapid genetic adaptation to warming in a Great Barrier Reef coral
Source: PLoS Genet. 2018 Apr 19;14(4):e1007220. doi: 10.1371/journal.pgen.1007220 (PMC5908067; doi:10.1371/journal.pgen.1007220)

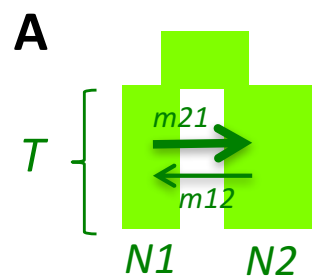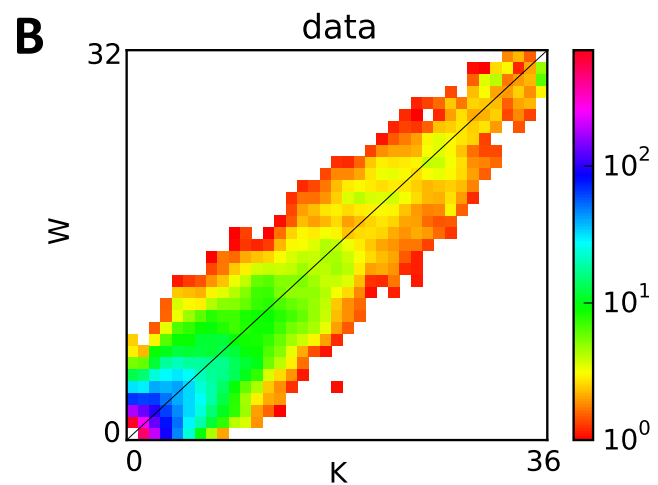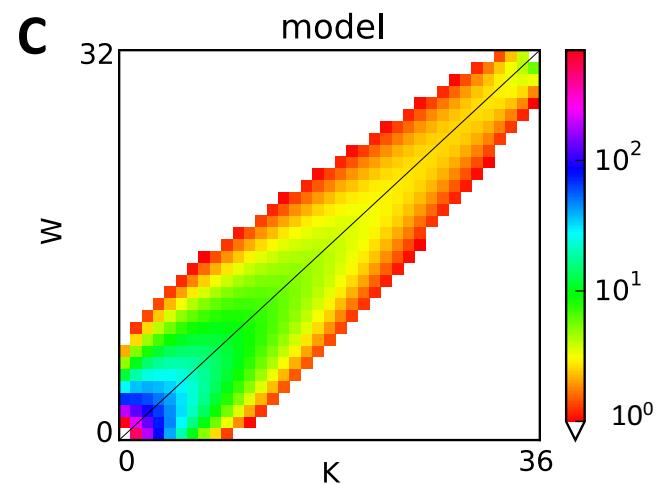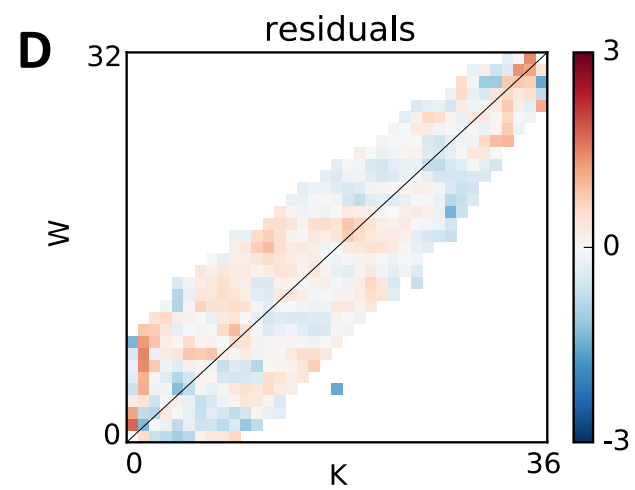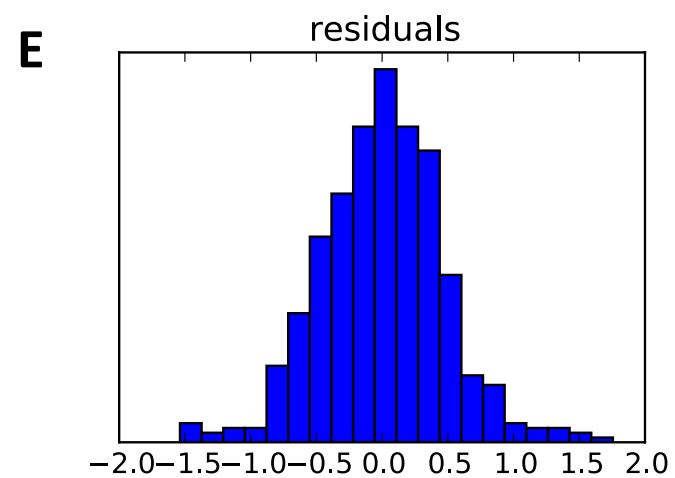

Supplement: S1 Fig — (A) The model: ancestral population splits into two populations of unequal sizes (N1 and N2) some time T in the past, which exchange migrants with different rates depending on direction. (B) Observed allele frequency spectrum comparing Wilkie (W) and Keppel (K) populations. (C) Allele frequency spectrum generated by the fitted model. (D, E) Map and histogram of residuals (absolute scale). (PDF) [file pgen.1007220.s002.pdf]

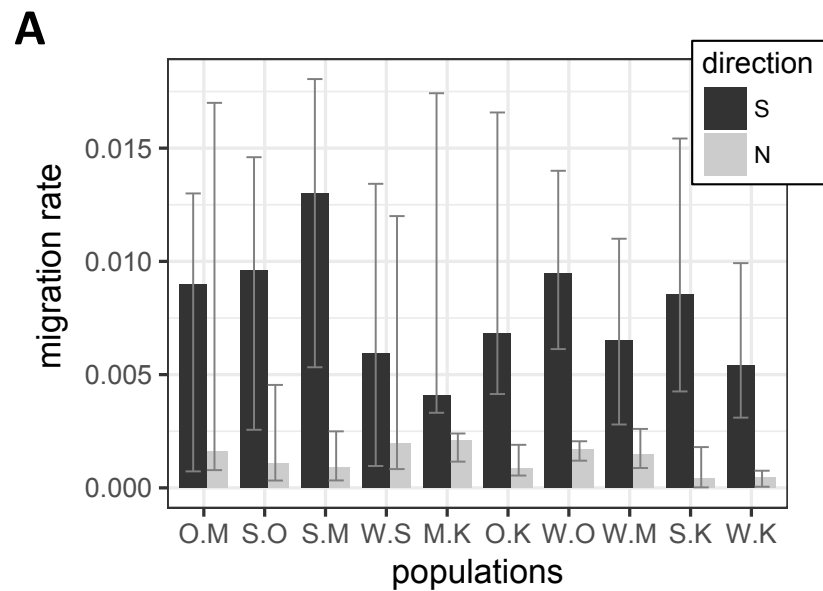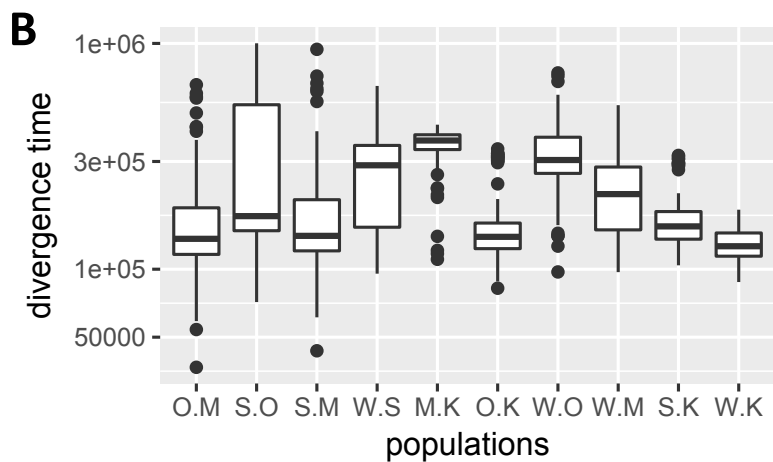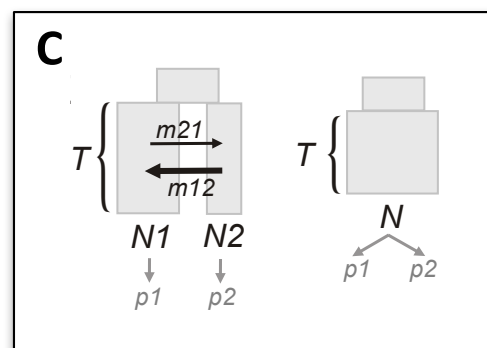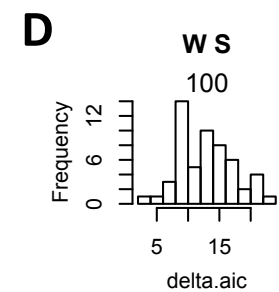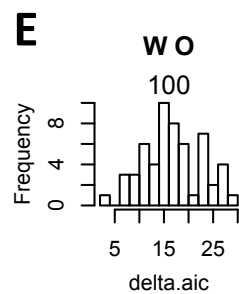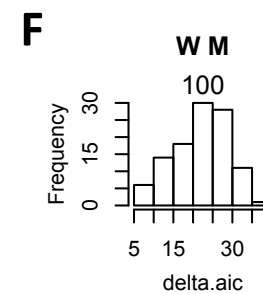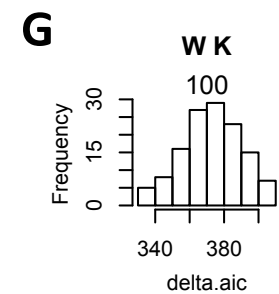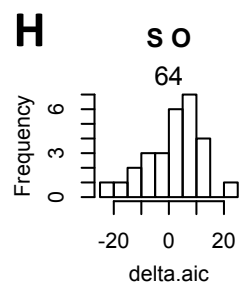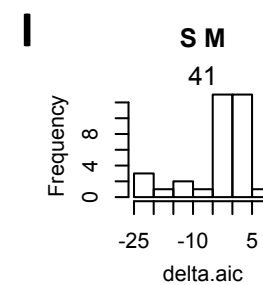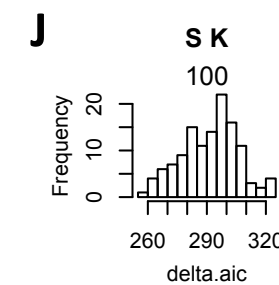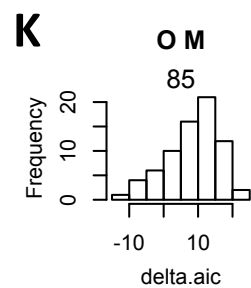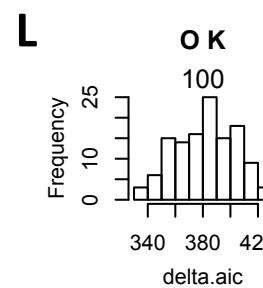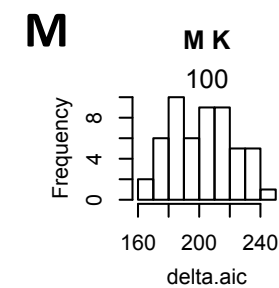

Supplement: S2 Fig — (A) Migration among population pairs, with bootstrap-derived 95% confidence intervals. The pairs are identified on the x-axis and sorted by increasing geographical distance. Black bars–southward migration, grey bars–northward migration. (B) Boxplot of divergence times (in years, y-axis) between pairs of populations (x-axis) across bootstrap replicates. (C) Models being compared: the split model (left) implies populations’ split into two different sizes (N1 and N2) at time T in the past, since when they exchanged migrants at unequal rates depending on direction. No-split model (right) allows for population size change at time T in the past but does not include population split: the two genotyped groups (p1 and p2) are regarded as two samples from the same population. (D-M) Histograms of delta-AIC values comparing split and no-split models (panel A) for bootstrap replicates (bootstrap was performed over genomic contigs of the draft genome of A. digitifera). Positive numbers indicate support for the split model. The letters on top of each panel identify compared populations, the number is the proportion of positive bootstrap replicates (i.e., bootstrap support for the full model). (PDF) [file pgen.1007220.s003.pdf]

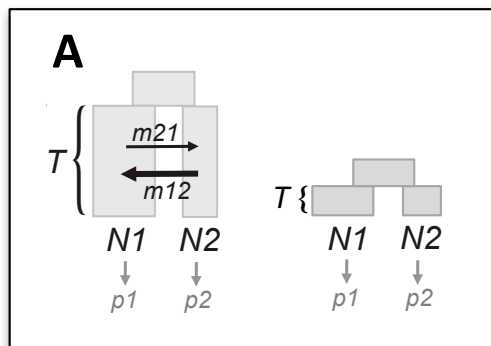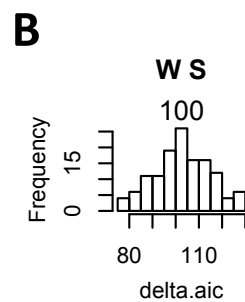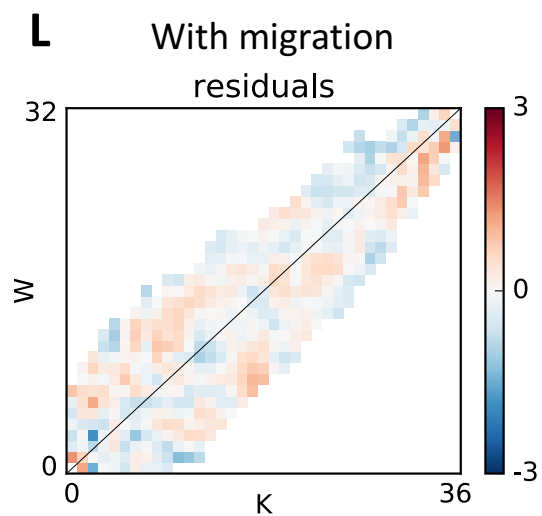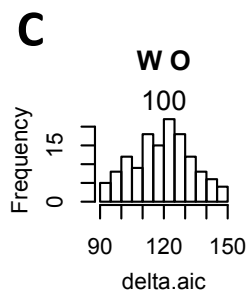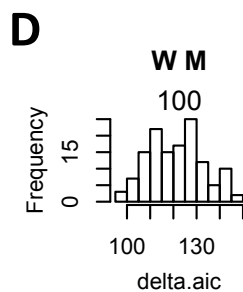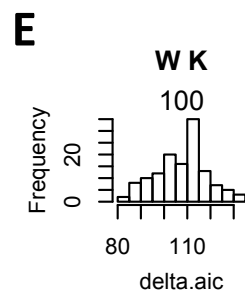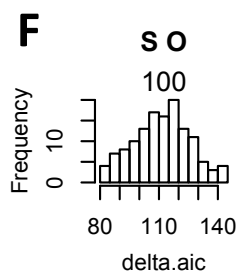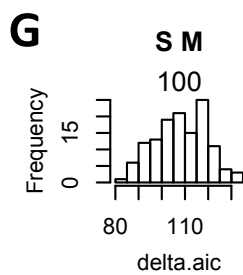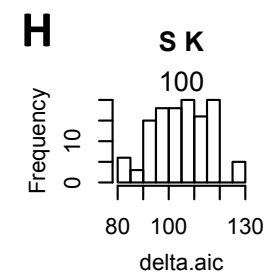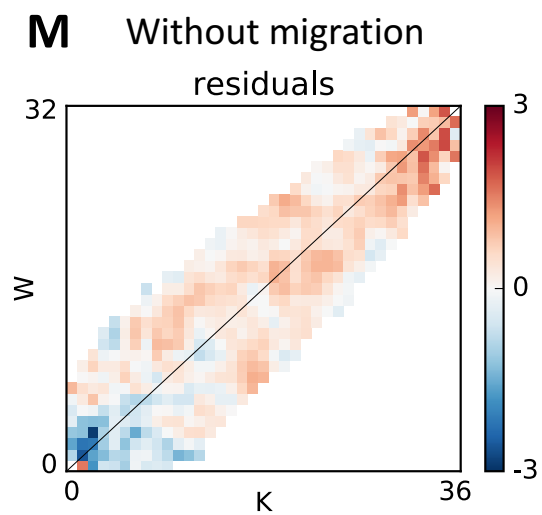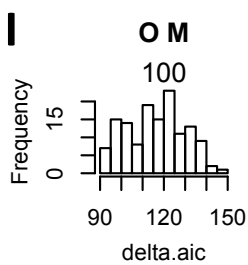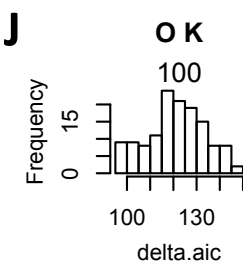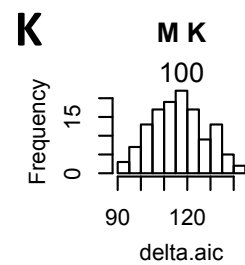

Supplement: S3 Fig — Delta-AIC bootstrap comparison of models with and without migration (A), to confirm that the model with migration and ancient divergence is preferable to the model with no migration but very recent divergence. (B-K) Histograms of delta-AIC values for bootstrap replicates comparing models with and without migration. Positive numbers indicate support for the model with migration. The letters on top of each panel identify compared populations, the number is the proportion of bootstrap replicates supporting the model with migration and ancient split. For all pairs of populations the model of ancient split with migration is strongly supported. (L, M) Example of residuals from the two models. Model without migration under-estimates the number of shared low-frequency polymorphisms and over-estimates the number of shared high-frequency polymorphisms. (PDF) [file pgen.1007220.s004.pdf]

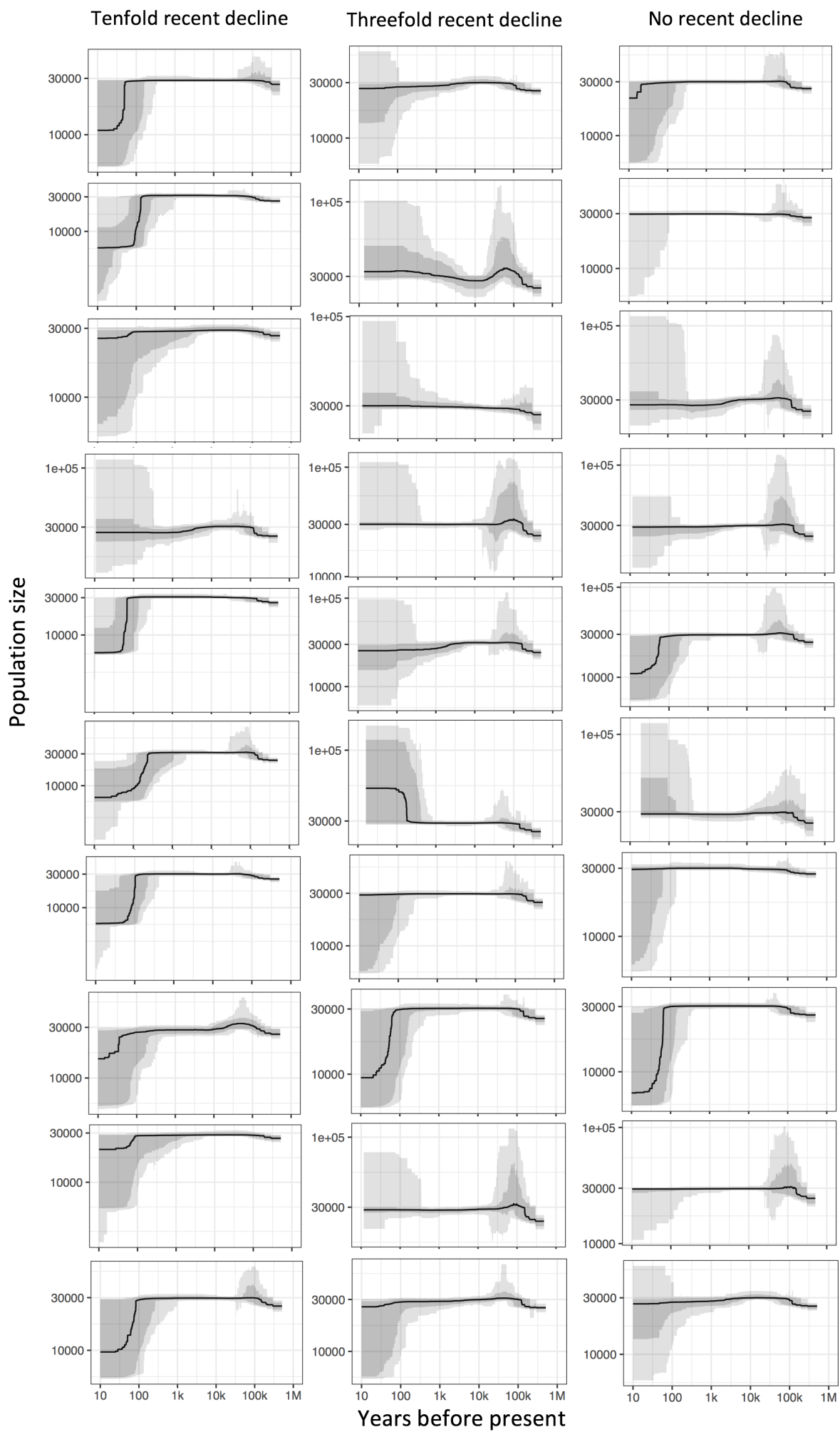

Supplement: S4 Fig — For each population size change scenario, ten datasets (evolution of 20,000 2bRAD loci in a population of 30,000 individuals) were simulated in SLiM. The line is median of 200 stairwayPlot bootstrap replicates for each of these datasets, light shaded area is 95% credible interval, dark-shaded area is 75% credible interval. Despite generally high uncertainty about recent population sizes, tenfold decline (left column) is detectable as a drop in the median in 9 out of 10 cases, although rarely at the 95% confidence level. Threefold decline scenario (middle column) does not show a pattern different from the no-change scenario (right column). (PDF) [file pgen.1007220.s005.pdf]

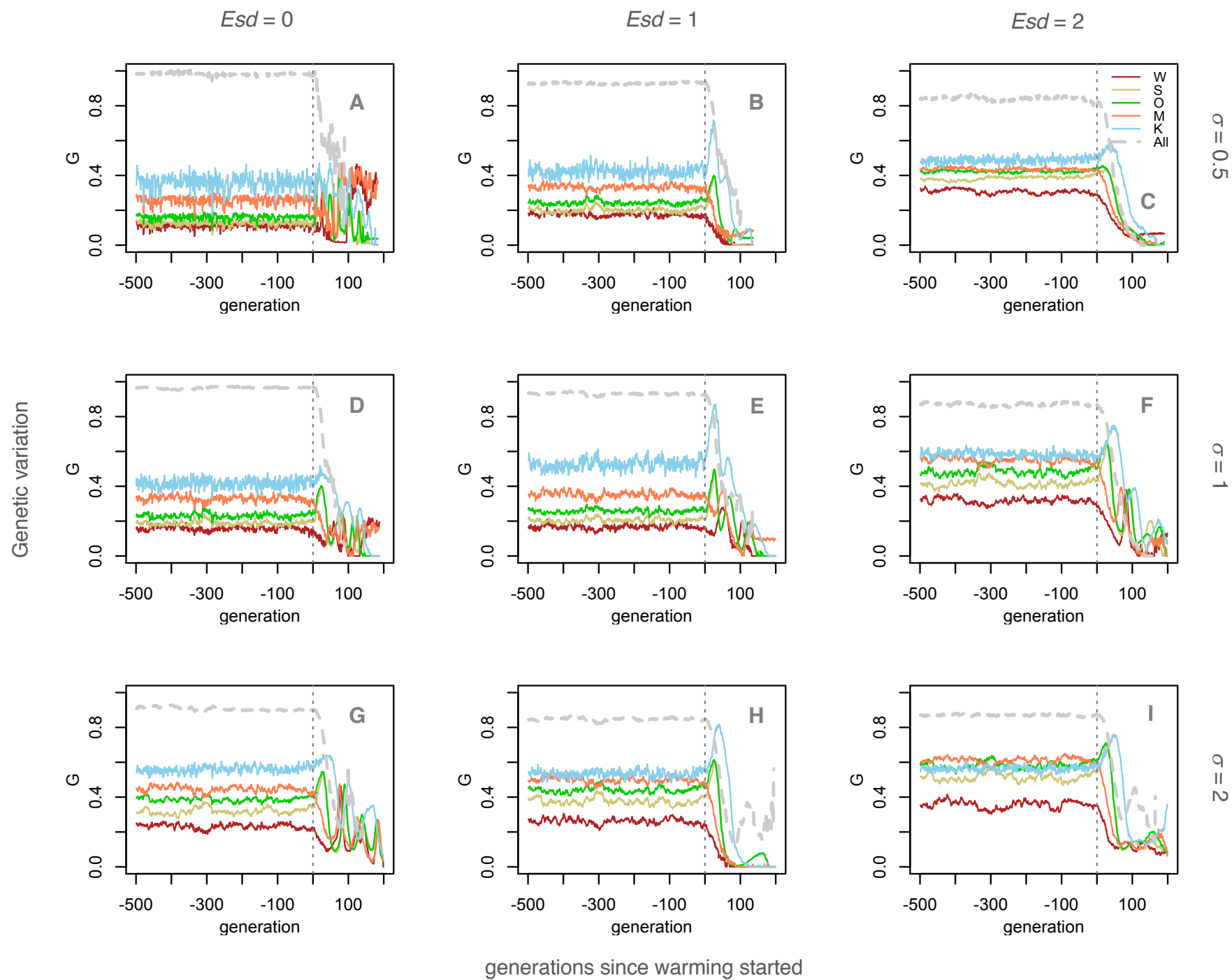

Supplement: S5 Fig — Total metapopulation genetic variation stays constant over the 500 generations preceding warming (up to generation 0), indicating genetic equilibrium. Note that at the settings for inefficient selection (high Esd and σ, panel H, F, and I) the variation within populations increases, especially at locations receiving many immigrants (all except W). In all cases, warming results in the rapid initial drop in total genetic variation. Genetic variation was calculated as standard deviation of breeding values across individuals. (PDF) [file pgen.1007220.s006.pdf]

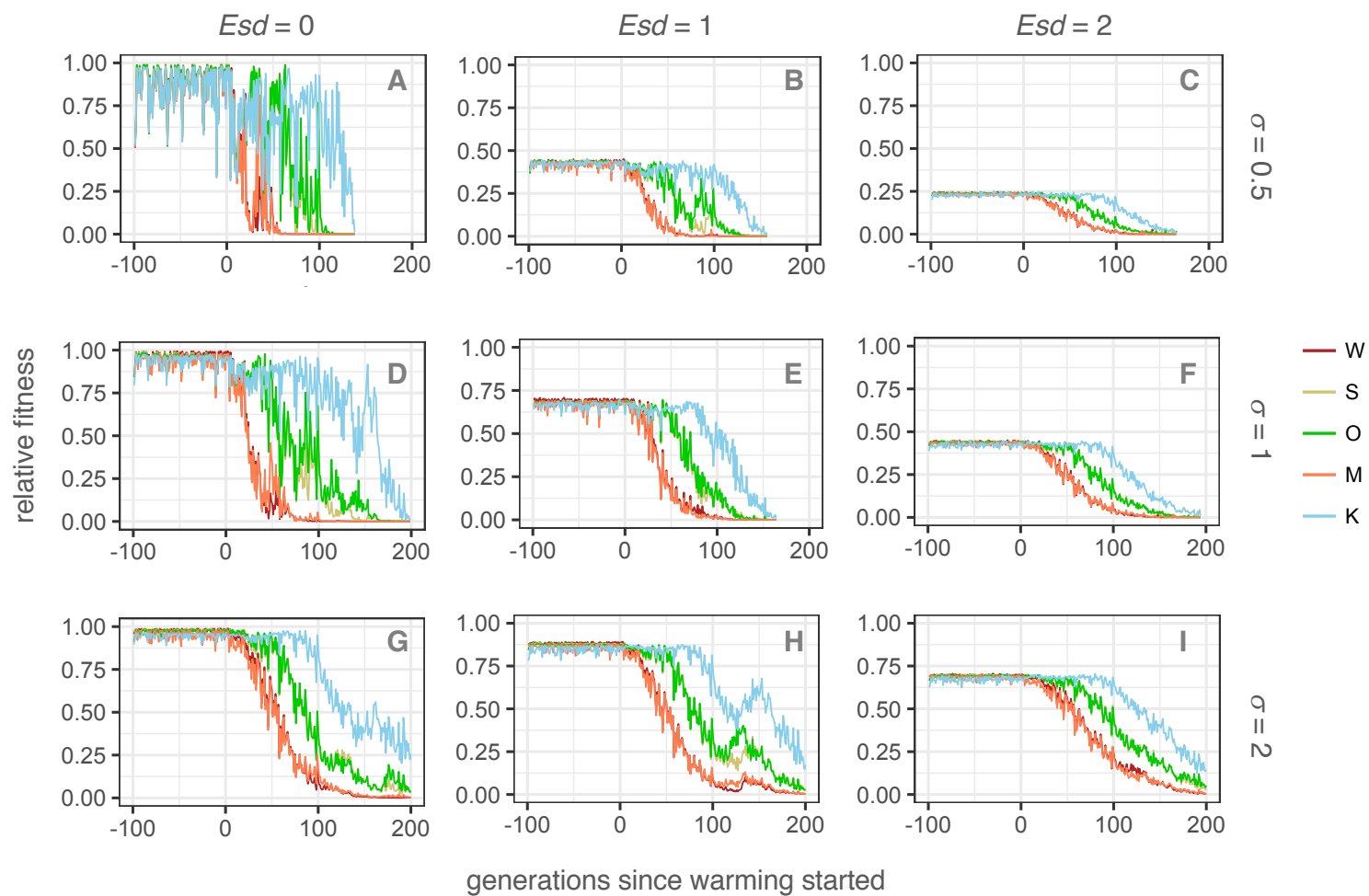

Supplement: S6 Fig — Rows of panels (A-C, D-F, G-I) correspond to different σ indicated to the right of the rows; columns of panels correspond to the same Esd value indicated above the column. Lower heritability strongly diminishes the fitness attained during pre-adaptation to local conditions, but mitigates the influence of random thermal anomalies. Broader tolerance also mitigates the effect of anomalies, partially rescues the drop in fitness due to low heritability, and promotes longer population persistence. (PDF) [file pgen.1007220.s007.pdf]

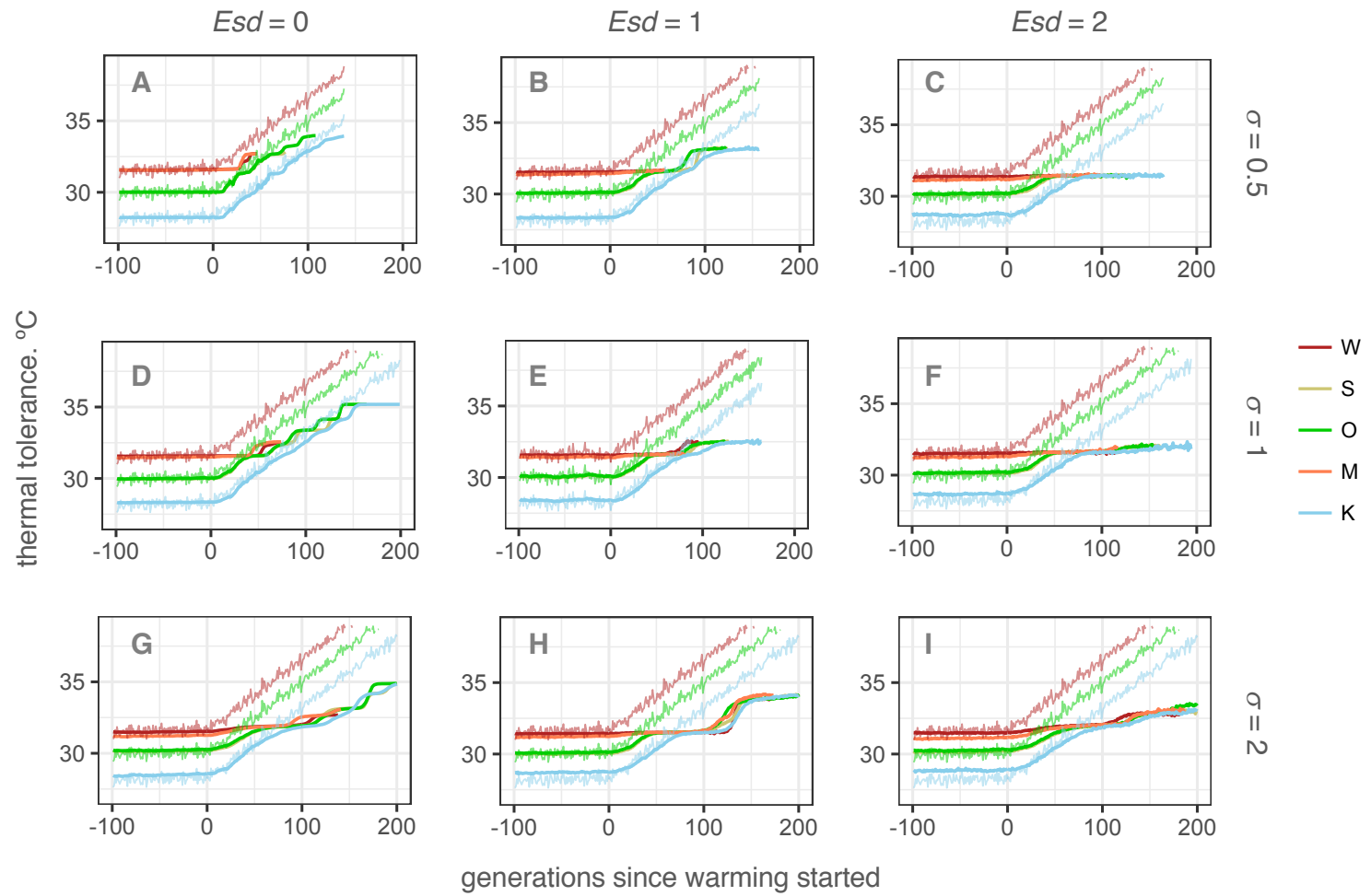

Supplement: S7 Fig — Rows of panels (A-C, D-F, G-I) correspond to different σ indicated to the right of the rows; columns of panels correspond to the same Esd value indicated above the column. Thin noisy lines show temperature in the environment. Lower heritability diminishes the efficiency of local adaptation and directed evolution without affecting long-term persistence. Broader thermal tolerance has the same effect on local adaptation and directed evolution but improves population persistence. (PDF) [file pgen.1007220.s008.pdf]
